# Targeted AAV5-Smad7 gene therapy inhibits corneal scarring *in vivo*

**DOI:** 10.1371/journal.pone.0172928

**Published:** 2017-03-24

**Authors:** Suneel Gupta, Jason T. Rodier, Ajay Sharma, Elizabeth A. Giuliano, Prashant R. Sinha, Nathan P. Hesemann, Arkasubhra Ghosh, Rajiv R. Mohan

**Affiliations:** 1 Harry S. Truman Memorial Veterans’ Hospital, Columbia, Missouri, United States of America; 2 Department of Veterinary Medicine & Surgery, College of Veterinary Medicine, University of Missouri, Columbia, Missouri, United States of America; 3 Mason Eye Institute, School of Medicine, University of Missouri, Columbia, Missouri, United States of America; 4 Chapman University School of Pharmacy, Irvine, California, United States of America; 5 GROW Research Laboratory, Narayana Nethralaya Foundation, Bangalore, Karnataka, India; Cedars-Sinai Medical Center, UNITED STATES

## Abstract

Corneal scarring is due to aberrant activity of the transforming growth factor β (TGFβ) signaling pathway following traumatic, mechanical, infectious, or surgical injury. Altered TGFβ signaling cascade leads to downstream Smad (Suppressor of mothers against decapentaplegic) protein-mediated signaling events that regulate expression of extracellular matrix and myogenic proteins. These events lead to transdifferentiation of keratocytes into myofibroblasts through fibroblasts and often results in permanent corneal scarring. Hence, therapeutic targets that reduce transdifferentiation of fibroblasts into myofibroblasts may provide a clinically relevant approach to treat corneal fibrosis and improve long-term visual outcomes. Smad7 protein regulates the functional effects of TGFβ signaling during corneal wound healing. We tested that targeted delivery of Smad7 using recombinant adeno-associated virus serotype 5 (AAV5-Smad7) delivered to the corneal stroma can inhibit corneal haze post photorefractive keratectomy (PRK) *in vivo* in a rabbit corneal injury model. We demonstrate that a single topical application of AAV5-Smad7 in rabbit cornea post-PRK led to a significant decrease in corneal haze and corneal fibrosis. Further, histopathology revealed lack of immune cell infiltration following AAV5-Smad7 gene transfer into the corneal stroma. Our data demonstrates that AAV5-Smad7 gene therapy is relatively safe with significant potential for the treatment of corneal disease currently resulting in fibrosis and impaired vision.

## Introduction

Homeostatic mechanisms in corneal tissue regulate different physiological responses including cell proliferation, migration, differentiation and apoptosis [1]. Corneal injury resulting from traumatic, mechanical, infectious, or surgical causes leads to the disruption of normal homeostatic processes, thereby inducing aberrant tissue remodeling with resultant corneal scarring. Corneal scarring is the third leading cause of blindness globally, affecting 1.3 million Americans each year [2,3]. Currently, no effective topical treatments exist that cure corneal scarring and the use of intra-operative drugs such as MMC (Mitomycin C) for prevention of corneal haze can be problematic [4]. Hence, corneal transplantation remains the gold standard for treatment of corneal blindness. However, limited availability of donor corneas and post-transplantation complications including drug-induced toxicity, immunosuppression, and graft failure and rejection limit the clinical success of corneal transplantation [5]. Therefore, there is an unmet clinical need to develop novel therapies for treatment of corneal disorders.

Gene therapy is one of the most promising approaches for the treatment of ocular surface disorders, including corneal scarring [6–10]. The cornea is an ideal target tissue for gene transfer due to its well-defined anatomy and easy accessibility [11] that permits direct topical instillation of gene delivery vectors. Treatment follow-up could be performed non-invasively using modalities such as slit-lamp biomicroscopy and high-resolution imaging using slit-lamp and stereo microscopes [12]. Different viral and non-viral vectors have been used to deliver therapeutic genes into the cornea *in vivo* [6–10,13–15]. Adeno-associated virus (AAV) is a small, nonpathogenic, single-stranded DNA virus of the parvovirus family that is a highly efficient vector for gene therapy applications [16]. We have successfully used multiple AAV serotypes for the efficient and safe delivery of therapeutic genes into rodent, rabbit, canine, equine, and human cornea [13,17–19].

Multiple growth factors and cytokines, including transforming growth factor β (TGFβ), released from corneal epithelium post injury play a critical role in the induction of corneal fibrosis by promoting myofibroblast formation as well as synthesis of extracellular matrix (ECM) and cytoskeletal proteins [9,14, 20]. TGFβ signaling plays a crucial role in transdifferentiation of keratocytes into myofibroblasts, the abnormal proliferation of which leads to aberrant collagen deposition causing fibrosis and ultimately loss of visual acuity [4, 14, 21]. Smad (Suppressor of mothers against decapentaplegic) proteins have been shown to be specifically activated by TGFβ superfamily members [22] and they regulate cell growth, proliferation, migration, and differentiation. Therefore, Smad proteins provide an attractive target for developing novel interventional strategies for corneal scarring [14]. Various Smad proteins mediate downstream signaling cascades during corneal wound repair processes, especially during the regenerative healing phase [23]. Smad7 negatively regulates TGFβ signaling by binding to the TGFβ type 1 receptor through its MH2 domain and competing with R-Smad-Smad4 complex for DNA binding [24–27]. Since Smad7 blocks epithelial to mesenchymal transition as well as transdifferentiation of fibroblasts into myofibroblasts, we hypothesized that AAV-mediated targeted delivery of Smad7 in the corneal stroma could effectively treat corneal fibrosis. Here, we investigated the potential therapeutic efficacy and safety of AAV5-Smad7 targeted gene therapy in an established high diopter photorefractive keratectomy (PRK) induced corneal fibrosis model in rabbits *in vivo*.

## Materials and methods

### Primary human corneal fibroblast culture

Primary human corneal fibroblast (HCF) cultures were established from donated human corneas procured from an eye bank (Saving Sight, Kansas City, MO, USA). Briefly, human corneal tissues were washed twice with serum free sterile minimum essential medium (MEM) culture medium (Thermo Fischer Scientific, Grand Island, NY, USA), epithelial and endothelial layers were gently removed with surgical blade and remaining tissue was sub-sectioned for explant culture. The convex surface of the stromal sections was placed face down onto a 10 cm^2^ tissue culture dish and incubated in a humidified 5% CO_2_ incubator at 37°C. The explants were cultured in MEM supplemented with 10% fetal bovine serum for approximately 20+ days by changing medium every second day. The primary fibroblasts were harvested by trypsinization, seeded at a density of 7.5x10^4^ cells/well in six-well plates, and allowed to reach 60–70% confluence for further experiments. To generate myofibroblast cultures, HCF were initially seeded at a density of 7.5x10^4^ cells/well in six-well plates in MEM containing 10% fetal bovine serum, and switched to serum-free medium for 8–12 h. Cultures were further incubated in serum free medium supplemented with TGFβ1 (5 ng/ml) for an additional 4 days. The cultures were fed with fresh serum-free medium and TGFβ1, every 24 h.

### RNAi-mediated loss-of-function studies

For the loss-of-function studies, transient knockdown of Smad7 gene expression was achieved using pre-validated siRNA (Ambion Life technologies, Grand Island, NY, USA). For the long-term knockdown of Smad7, the pre-validated short hairpin RNA (shRNA) oilgos were cloned into a pcDNA 6.2 miR RNAi expression vector under the control of Pol II promoter (Life Technologies, Grand Island, NY, USA). The RNAi and shRNA plasmid transfections were performed using Lipofectamine3000 (Life technologies corporation, Grand Island, NY, USA) as per the manufacturer’s instructions. Briefly, a transfection solution was prepared by adding 10μl Lipofectamine 3000 in 200 μl of opti-MEM medium to pre-diluted RNAi/plasmid-RNAi in 200 μl of opti-MEM. The mixture was incubated for 10 min at room temperature and added to the HCF in 1.6 ml of a serum-free culture medium. The transfected cells were washed 16 h later and used for the loss-of-function studies.

### Generation of AAV5-Smad7

The Smad7 gene was PCR-amplified from rabbit corneal fibroblast cDNA and cloned into the AAV5 plasmid pTRUF11. The expression cassette contained a hybrid promoter (cytomegalovirus enhancer and chicken *β*-actin promoter) and the simian virus 40 polyadenylation site. Multiple clones were sequenced to rule out any potential mutations introduced during PCR. Recombinant AAV5-Smad7 virus was generated using a two-plasmid packaging system as previously reported [8].

### Animal studies

Two to three month-old New Zealand White female rabbits (Covance Research Products, Denver, PA, USA) weighing 2.5 to 3.0 kg were used for the gene therapy study. The Institutional Animal Care and Use Committee of the University of Missouri, Columbia and Harry S. Truman Memorial Veterans’ Hospital, Columbia, Missouri approved the animal study. All procedures and animals were treated in accordance with the ARVO Statement for the Use of Animals in Ophthalmology and Vision Research. Anesthesia was performed in rabbits by intramuscular injection of a cocktail containing ketamine hydrochloride (50 mg/kg) and xylazine hydrochloride (10 mg/kg). Topical ophthalmic anesthesia solution, proparacaine hydrochloride, was applied prior to any procedure.

### Intraocular pressure (IOP) measurement by tonometry

Intraocular pressure (IOP) recordings, while commonly recognized as altered in intraocular diseases such as glaucoma (above normal reference ranges) and uveitis (below normal reference ranges) in the clinical patient, may be influenced by corneal thickness, rigidity, and abnormalities in corneal anatomy. A significant potential concern with any ocular gene therapy is vector delivery associated ocular pathophysiological changes, including aqueous humor regulation. Therefore, as an additional means to ensure that neither the cornea, nor normal intraocular fluid dynamics, were not being adversely affected by any of our treatments. IOP of both eyes in all study rabbits was recorded using a TONO-PEN (Reichert TONO-PEN AVIA tonometer, NY, USA) at day 0, 7, 14, 21 and 28 days. All IOP measurements were performed between 9-11am to minimize normal diurnal variations in IOP.

### *In vivo* model for corneal scarring

Corneal fibrosis was induced in rabbit corneas using high diopter (–9D) photorefractive keratectomy (PRK) after epithelial-debridement as described previously [28]. Only one eye of each animal was used for PRK while the other eye served as contralateral untreated control. Briefly, in an anesthetized rabbit, a wire eyelid speculum (10×4 mm) was inserted to expose the corneal surface for treatment and 2 to 3 drops of proparacaine hydrochloride solution was topically applied. Thereafter, corneal epithelium was debrided by gentle scraping with #64 Beaver blade (BD Biosciences; Franklin Lakes NJ, USA). PRK surgery was performed by creating a 6-mm ablation zone on the central stroma using laser pulses for –9D treatment with an excimer laser (Summit Apex; Alcon Orlando FL, USA), to produce fibrosis (haze) in the cornea. This surgery is known to produce consistent fibrosis and fibroblast to myofibroblast conversion in the rabbit cornea that peaks at 4 weeks [8].

### AAV5-Smad7 transduction into rabbit cornea

The rabbits were divided into two groups. In group-I (treatment arm), 75μL of AAV5-Smad7 titer (2.67 × 10^13^ μg/mL; *n* = 6) was topically applied to the rabbit cornea for 5 minutes via a custom cloning cylinder technique, immediately after PRK surgery [7, 8]. In group-II (corresponding control arm), rabbit corneas received 75μL of AAV5-naked vector titer (titer 1.0 × 10^13^ μg/mL; *n* = 6) topically for 5 minutes via a cloning cylinder right after PRK surgery in a similar fashion as group-I animals [7, 8]. The contralateral corneas served as naïve/untreated control (no PRK, no AAV titer and no epithelial-debridement; n = 6). For each group, slit- lamp biomicroscopy was performed in all rabbit eyes at regular intervals (day 0, 7, 14, 21 and 28).

### Corneal haze and analysis through biomicroscopy *in vivo*

The overall health of the rabbit corneas and degree of corneal haze was evaluated prior to initiating the study and at regular intervals using a slit-lamp biomicroscope (SL-15 portable slit lamp, Kowa Optimed Inc. Torrance, CA, USA) and imaged at 4 weeks after PRK with a coupled digital imaging system as previously reported [8, 28]. Haze scoring was performed according to the Fantes grading scale [29, 30]. Briefly, grade 0 was a completely clear cornea; grade 0.5 had trace haze visualized with careful tangential illumination on slit-lamp biomicroscopy; grade 1 was more prominent haze not interfering with the visibility of fine iris details; grade 2 represented sufficient haze as to cause mild obscuration of iris details; grade 3 was moderate obscuration of the iris and lens; and grade 4 was complete opacification of the cornea in the area of ablation.

### Corneal tissue collection

Following the completion of the experimental study, the rabbits were humanely euthanatized with a pentobarbital (150 mg/kg) overdose while under general anesthesia. Corneas of rabbits were removed with forceps and sharp Westcott scissors, embedded in liquid optimal cutting temperature (OCT) compound (Sakura FineTek, Torrance, CA, USA) in a 24×24×5 mm mold (Fisher Scientific, Pittsburgh, PA, USA) and snap frozen [28]. Frozen tissue blocks were maintained at -80°C until further processed. Tissue sections were cut 8 μm thick with a cryostat (HM525 NX UV; Microm GmbH, Waldorf, Germany), placed on 25 × 75 × 1 mm microscope slides (Superfrost Plus; Fisher Scientific, Pittsburgh, PA, USA) and maintained frozen at -80°C until staining. For DNA preparation, corneal tissue samples were stored in cryo-vials and placed immediately into liquid nitrogen for further processing and examination.

### TUNEL assay and immunofluorescence studies

Cryo-sections of corneal tissue blocks were used for immunofluorescence studies. Briefly, tissue sections were incubated with 2% bovine serum albumin for 30 minutes at room temperature and then with respective primary antibodies. After primary antibody staining, the sections were exposed to Alexa 594 donkey anti-goat IgG secondary antibody (1:500 dilution, A11058; Invitrogen) for 1 h in the dark. Myofibroblasts were identified in tissues by staining for α-smooth muscle actin (α-SMA) using a mouse monoclonal antibody (1:200 dilution, M0851; Dako, Carpinteria, CA, USA) for 90 minutes. The α-SMA^+^ cells were quantified in six randomly selected, non-overlapping, full-thickness central corneal columns, extending from the anterior to the posterior stromal surfaces [8, 28, 30]. Filamentous actin (F-actin) staining was performed with Alexa 594 conjugated phallotoxin (A12381; Invitrogen). The tissues were incubated with 1% bovine serum albumin for 30 minutes at room temperature and then with Alexa 594 conjugated phallotoxin at 1:40 dilution for 30 minutes in the dark followed by subsequent washing with PBS. For quantification, 200 and 400 × magnification field were used for α-SMA and F-actin respectively. Fibronectin was stained using goat polyclonal primary antibody (1:200 dilution, sc6952; Santa Cruz Biotechnology Inc., Santa Cruz, CA, USA). After primary antibody incubation, tissue sections were exposed to Alexa 594 donkey anti-goat IgG secondary antibody (1:500 dilution, A11058; Invitrogen) for 1 h in the dark.

TUNEL assay was performed according to manufacturer’s instructions (ApopTag; Millipore). In brief, the rabbit corneal tissue sections were fixed in 1% paraformaldehyde at room temperature followed by subsequent permeabilization with ethanol: acetic acid (2:1 ratio; at -20°C), and treated with fluorescent detection assay kit reagents to detect apoptosis and/or necrosis.

### RT-PCR analysis to determine gene copy number

The AAV5-Smad7 gene copy number in corneal tissues was determined by quantitative-PCR analysis. The frozen corneal tissues were ground in liquid nitrogen and DNA was isolated (DNeasy kit; Qiagen, Valencia, CA, USA). The standard curves were plotted using a 10-fold serial dilution of Smad7 plasmid vector (10^4^ to 10^9^). For the PCR reaction, forward (5’ CTC CAT CAA GGC TTT CGA CTA C 3’) and reverse (5’ AGC TGA TCT GCA CGG TAA AG 3’) primers were used with the following cycling conditions: 95°C for 10 min, 40 cycles at 95°C for 15 s, and 60°C for 60 s. The AAV5-delivered Smad7 gene copies were calculated in per mg of DNA as previously reported [30].

Rabbit frozen corneal tissue sections were used for Smad7 immunofluorescence to verify endogenous and AAV5-delivered Smad7 expression. Briefly, rabbit tissue sections were incubated with 2% bovine serum albumin for 30 minutes at room temperature followed by Smad7 primary antibodies incubation (1:100 dilution, sc101152; Santa Cruz Biotechnology Inc., Santa Cruz, CA CA). After this, sections were incubated with Alexa 594 donkey anti-mouse IgG secondary antibody (1:500 dilution, A21203; Invitrogen) for 1 h in dark.

### Statistical analyses

The results of corneal haze grading and other quantification studies were expressed as the mean ± SEM. Statistical analysis was performed with student’s *t*-test or the Wilcoxon rank sum test, One-way analysis of variance (ANOVA), and Bonferroni test. *P* < 0.05 or 0.005 indicated significance in different experiments.

## Results

### TGFβ1 induced myofibroblast induction is not affected by Smad7 loss-of-function

**Fig 1:**
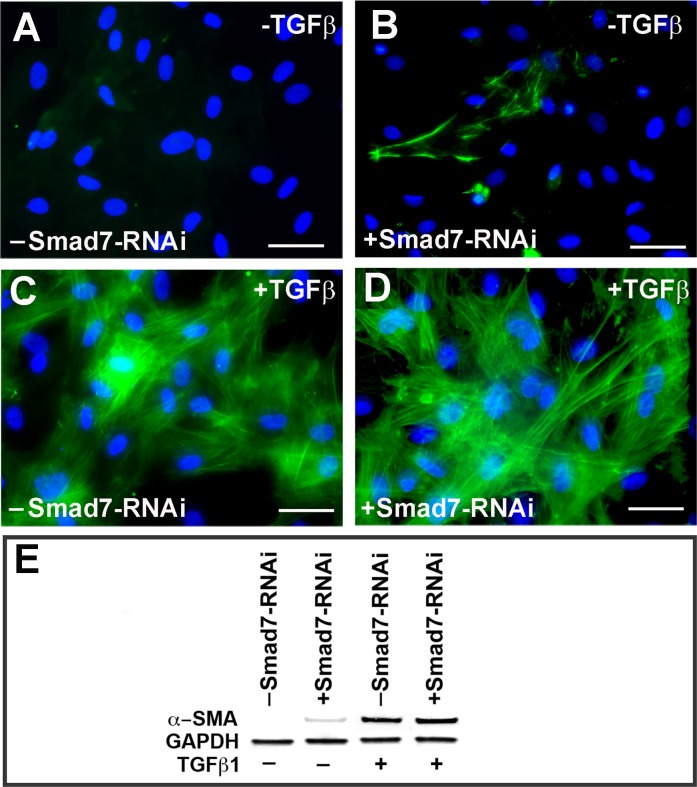
Smad7 loss augments fibrosis in TGFβ1 treated HCF. Effect of Smad7 knockdown on TGFβ1 mediated HCF trans-differentiation to myofibroblasts was evaluated *in vitro*. α-SMA immunostaining in mock treated HCF (A), Smad7-siRNA transfected and no TGFβ1 (B), TGFβ1 treated HCF (C), Smad7-siRNA and TGFβ1 treated (D) and immunoblotting (E) showed α-SMA levels for indicated treatments; GAPDH serves as loading control.

We hypothesized that overexpression of Smad7 will abrogate TGFβ-mediated transdifferentiation of keratocytes into myofibroblasts thereby preventing corneal fibrosis *in vivo*. To test our hypothesis, we first performed RNAi mediated gene silencing studies to knockdown the expression of Smad7 *in vitro* using TGFβ1 treated HCF (Fig 1). Our prior studies revealed minimal α-SMA immunostaining in HCF transfected with Smad2, Smad3 or Smad4 [31]. We observed that despite RNAi-mediated knockdown of Smad7, TGFβ1 treated HCF resulted in 95% of the cells being α-SMA+ (p<0.001), similar to TGFβ1 treated control HCF (Fig 1C and 1D). In contrast, lack of TGFβ1 treatment failed to upregulate α-SMA staining in the control HCF (Fig 1A), but there were a few α-SMA+ cells in the siSmad7 treated group without TGFβ1 treatment (Fig 1B). We further confirmed our loss-of-function results by western blot analysis (Fig 1E) that shows α-SMA induction by TGFβ1 treatment in the control and Smad7-RNAi transfected cells.

### Smad7 upregulation via AAV5 gene transfer inhibits myofibroblast differentiation

**Fig 2:**
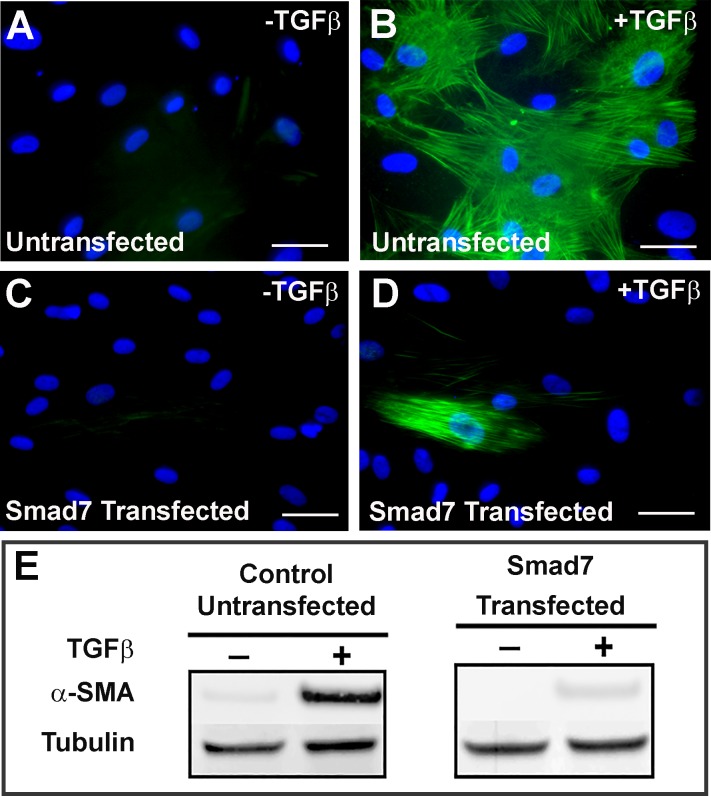
Smad7 gain-of-function inhibits fibrosis in TGFβ1 treated HCF. Smad7 overexpression was studied by transfecting HCF with Smad7 overexpressing vector and growing with or without TGFβ1. Images showing α-SMA levels in non-transfected without TGFβ1 negative control (A), non-transfected and TGFβ1 treated positive control (B), Smad7 transfected and no TGFβ1 treated (C) and Smad7 transfected and TGFβ1 treated (D) HCF. Immunoblot analysis (E) for α-SMA levels in HCF with indicated transfections and treatments. Tubulin serves as loading control.

To validate our lead hypothesis, we tested Smad7 overexpression by transient transfection of Smad7 expressing plasmid in HCF cultures. Our α-SMA immunostaining data and western blot analysis revealed that transient Smad7 overexpression significantly reduced α-SMA levels (93%; p<0.001) without altering the phenotype or cell viability (Fig 2A–2D). This reduction in α-SMA positive myofibroblasts was observed even in TGFβ treated cells (Fig 2D). The western blot analysis of non-transfected HCF revealed significant upregulation of α-SMA in the presence of TGFβ that was inhibited by Smad7 overexpression (Fig 2E).

### AAV5-Smad7 gene transfer reduces corneal haze in post PRK in rabbit cornea

To test the potential therapeutic efficacy of Smad7 *in vivo* for treating corneal fibrosis, we developed a recombinant AAV5 expressing human Smad7 (AAV5-Smad7). Therapeutic efficacy of AAV5-Smad7 gene transfer was evaluated in rabbit eyes in a PRK induced corneal haze model. The rabbit corneas were treated with AAV5-Smad7 or AAV5-naked vector immediately after PRK. The corneal haze was examined 4 weeks post treatment using slit-lamp biomicroscopy and stereo-microscopy and graded according to Fantes scoring system [29].

**Fig 3:**
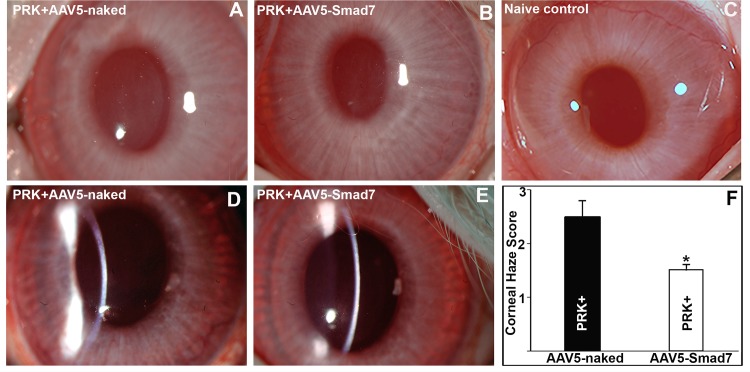
AAV5-Smad7 reduces corneal hazing *in vivo* post PRK. Representative slit lamp wide beam (A, B, C) and narrow beam (D, E) biomicroscopy images showing corneal haze in AAV5-naked vector no-gene delivered (A, D), AAV5-Smad7 gene delivered (B, E) and naïve (C) rabbit eyes. Graph (F) shows quantification of corneal haze.

As compared to the naïve group (Fig 3C), slit-lamp and stereo microscopic images revealed corneal haze post-PRK in both the AAV5-naked vector and AAV5-Smad7 gene delivered group (Fig 3A, 3B, 3D and 3E). PRK treated rabbit cornea without Smad7 gene delivery showed prominent haze and cloudiness because of strong fibrotic response after surgery (Fig 3A and 3D). However, rabbit corneas treated with AAV5-Smad7 post-PRK surgery showed significantly less pro-fibrotic response indicated by comparatively less cloudiness and haze (Fig 3B and 3E). The naïve control group (without PRK & without AAV5-Smad7) demonstrated normal corneal anatomy and transparency (Fig 3C). Haze quantification was performed by an observer masked to the treatment groups and revealed a significantly higher (p < 0.01) average haze score of 2.6 ± 0.5 in PRK only eyes compared to PRK with AAV5-Smad7 treatment eyes (1.4 ± 0.4) (Fig 3F). Furthermore, the results of slit-lamp biomicroscopy examination revealed that localized topical delivery of AAV5-Smad7 did not negatively alter corneal morphology or have any clinically noticeable adverse effects on corneal physiology. Subjective clinical evaluation on days 3, 7, 14, 21 and 28 found no evidence of intraocular inflammation, redness, ocular discharge, corneal or conjunctival edema, or infection in rabbit eyes. Taken together, our slit-lamp biomicroscopy and stereomicroscopy data suggest that AAV5-Smad7 gene transfer reduces corneal haze post-PRK in the rabbit cornea.

### AAV5-Smad7 gene therapy maintains physiological intraocular pressure (IOP)

**Fig 4:**
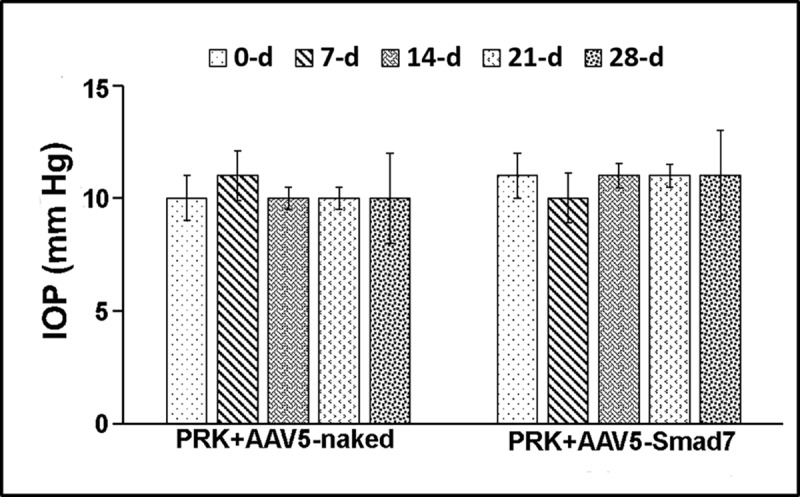
AAV5-Smad7 gene transfer does not alter intraocular pressure (IOP). IOPs were recorded on days 0, 7, 14, 21 and 28 post PRK in AAV5-naked or AAV5-Smad7 treatment using TONO-PEN method. An average of 3 readings per eye was used.

A major concern and a potential challenge with any ocular gene therapy is vector delivery associated ocular pathophysiological changes, including aqueous humor regulation. Inappropriately elevated IOP will cause glaucomatous damage over time. Alternatively, inappropriately low IOP results in ocular hypotony, with associated macular and corneal dysfunction. Here we determined IOP at days 0, 7, 14, 21 and 28 days in naïve, AAV5-naked vector as well as AAV5-Smad7 treated animals. The IOP data across the cohort did not show any significant changes during the entire course of the study (Fig 4).

### AAV5-Smad7 gene therapy effectively reduces scar formation

The presence of myofibroblasts is a characteristic feature of tissue fibrotic response including refractive surgery induced corneal haze. Typically, these myofibroblasts express bundles of filamentous proteins and therefore can be readily detected by staining for α-SMA and F-actin [32]. Increased fibronectin levels during corneal wound healing and fibronectin matrix assembly has been shown to facilitate TGFβ mediated myofibroblast induction [33]. To further evaluate the therapeutic efficacy of AAV5-Smad7 gene therapy, we performed immunohistochemical analysis of rabbit corneas that were treated with either AAV5-naked vector or AAV5-Smad7.

**Fig 5:**
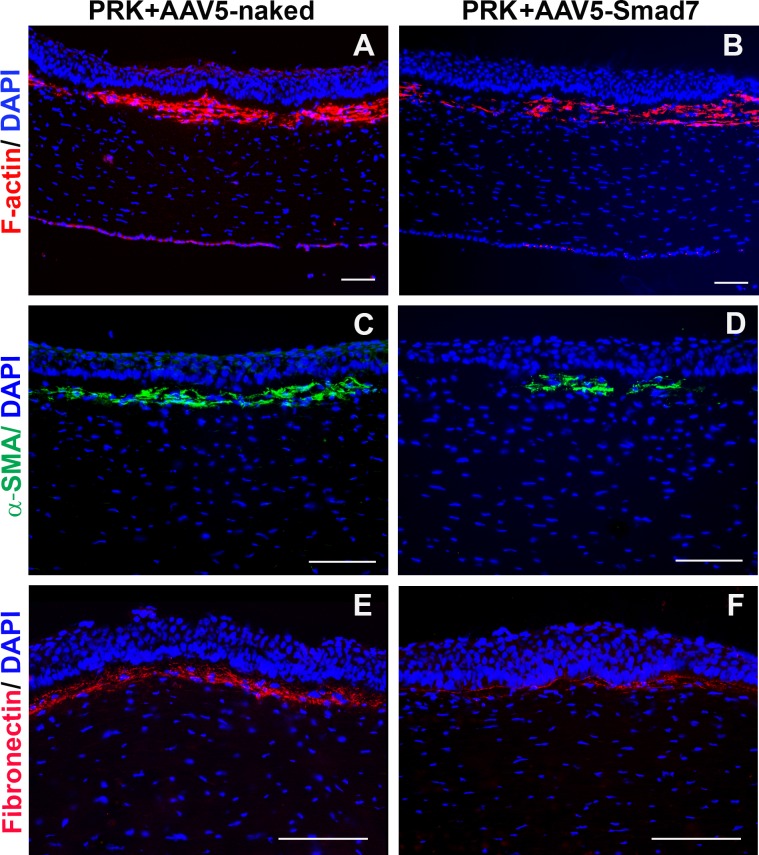
AAV5-Smad7 gene transfer reduces TGFβ-mediated corneal fibrosis. The images illustrate levels of profibrotic proteins in PRK-applied and AAV5-naked or AAV5-Smad7 treated eyes. Immunostaining for F-actin (A, B), α-SMA (C, D) and fibronectin (E, F) respectively. Scale Bar: 100 μm.

Tissue sections of rabbit corneas obtained 4 weeks after PRK surgery showed substantial α-SMA and F-actin positive staining just beneath corneal epithelium in the stroma confirming the formation of myofibroblasts (Fig 5). Immunostaining of F-actin (Fig 5A and 5B), α-SMA (Fig 5C and 5D), and fibronectin (Fig 5E and 5F) represents the comparative expression of these proteins in both AAV5-naked and AAV5-Smad7 groups respectively. No positive staining of the tested proteins was observed in naïve rabbit corneas (data not shown).

**Fig 6:**
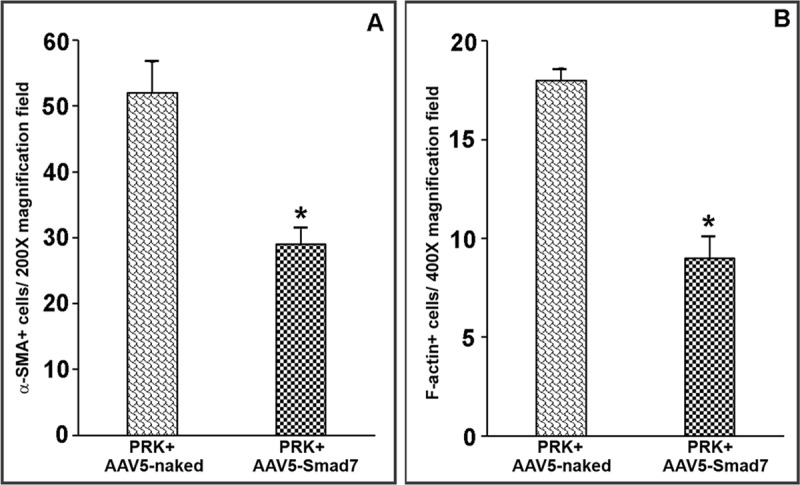
AAV-Smad7 inhibits α-SMA and F-actin expression *in vivo*. Quantitative analysis of α-SMA (A) and F-actin (B) positive cells per 200 and 400 X magnification frame respectively.

Quantitative immunohistochemical analysis was performed by counting the average number of α-SMA/F-actin positively stained cells per 200 and 400X magnification field in the corneal tissue sections of all groups. The cumulative quantitative data reveal a significant decrease in α-SMA (Fig 6A) (48%; p < 0.01) as well as F-actin (Fig 6B) (52%; p < 0.001) in AAV5-Smad7 treated rabbit corneas. Taken together, the anti-fibrotic effect of the Smad7 gene transfer showed similar effect on all three fibrotic response markers.

### AAV5-Smad7 gene transfer is non-cytotoxic to rabbit corneal tissue

**Fig 7:**
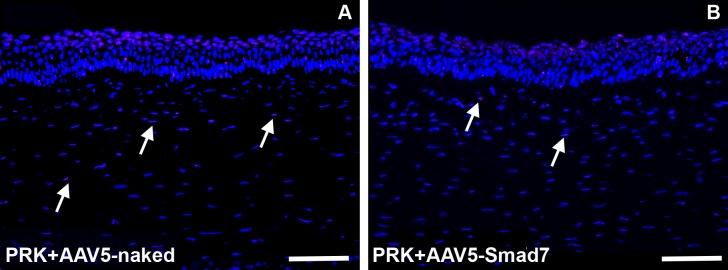
AAV5-Smad7 gene therapy is safe and nontoxic. TUNEL assay in rabbit AAV5-naked (A) and AAV5-Smad7 treated (B) cornea to demonstrate cellular toxicity and apoptotic process induced by gene transfer after 4-weeks. Scale Bar: 100 μm.

To study *in vivo* cytotoxicity mediated by AAV5-Smad7 treatment, we performed TUNEL assay on treated rabbit corneas. TUNEL immunostaining of rabbit cornea in AAV5-Smad7 vector showed the presence of three to five TUNEL^+^ cells in the stroma of both AAV5-Smad7 treated as well as AAV5-naked vector groups (Fig 7). Compared to the stroma, the corneal epithelium of both AAV5-Smad7 treated and AAV5-naked vector groups showed relatively higher number of TUNEL^+^ cells. The presence of higher number of TUNEL^+^ cells in the corneal epithelium indicates the normal turnover of the corneal epithelial cells. The TUNEL data suggests that AAV5-Smad7 is non-cytotoxic and safe for corneal gene delivery.

### AAV5-Smad7 gene transfer analysis

**Fig 8:**
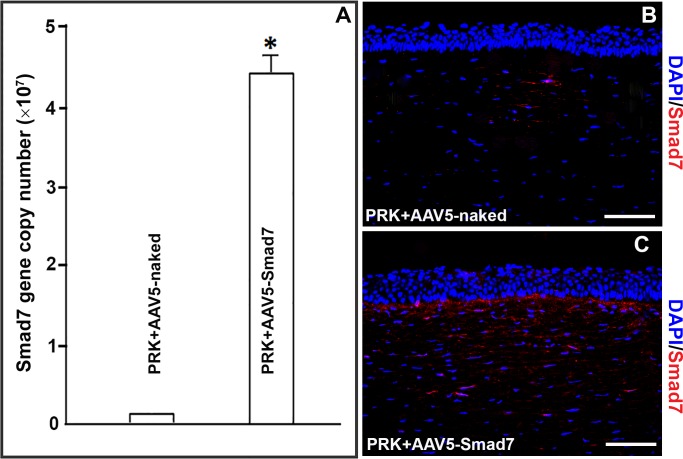
AAV5-Smad7 mediates high efficiency gene transfer in rabbit cornea. Quantitative RT-PCR (A) and qualitative Immunofluorescences (B, C) analyses were done for measuring AAV5-delivered Smad7 gene expression in AAV5-naked and AAV5-Smad7 treated rabbit corneal tissues post PRK.

High efficiency gene transfer is an essential prerequisite for successful therapy. The exogenous AAV5-Smad7 gene delivery after a single topical application on cornea was determined by quantitative PCR to quantify the Smad7 gene copy number. The data shown in Fig 8A, indicates that an average of 4.6 × 10^7^ genomic copies of Smad7 gene was delivered using AAV5-Smad7 vector in corneal tissue through our topical delivery method.

Fig 8B and 8C shows endogenous and delivered-Smad7 expression in the tissue sections of rabbit corneas obtained four weeks after PRK. Substantially increased Smad7-positively stained cells in the stroma of AAV5-Smad7 treated group (Fig 8C) compared to AAV5-naked group (Fig 8B) verified Smad7 gene delivery via AAV5.

### AAV5-Smad7 gene transfer molecular mechanism

**Fig 9:**
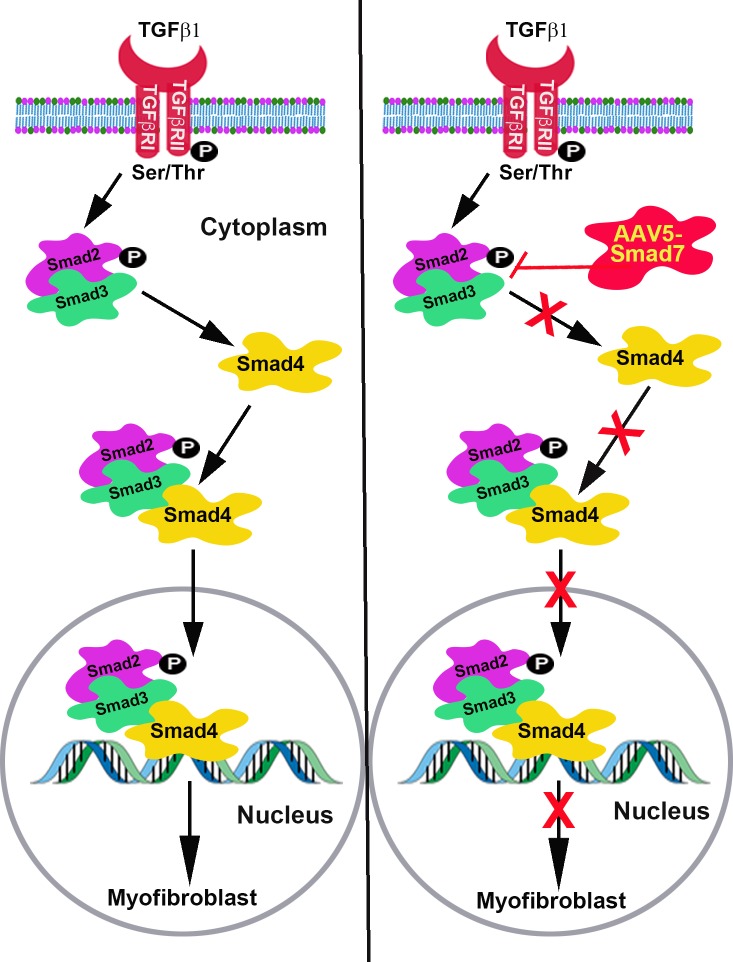
AAV5-Smad7 gene transfer hypothesis. Schematic representation of corneal fibrosis inhibition by AAV5-Smad7 gene transfer.

AAV5-Smad7 gene transfer attenuates corneal fibrosis by inhibiting TGFβ and Smad singling. The overexpression of Smad7 gene inhibits the formation of Smad2/3 and Smad4 complex formation and attenuates the trans-localization of Smad2/3 & Smad4 from cytoplasm to nucleus that regulate the transcription of myofibroblast formation (Fig 9).

## Discussion

Fibrosis is the leading cause of corneal blindness and can potentially be treated by targeted gene therapy [4,7,10]. Following corneal injury, TGFβ drives pro-fibrotic responses by enhancing ECM gene expression, and is responsible for the conversion of fibroblasts into myofibroblasts [34]. The activity of TGFβ initiated signaling pathways is under tight control by processes including regulation of the ligands, receptors and the key downstream intracellular effectors, of the Smad protein family [23–25]. Smad7 gene transfer has been demonstrated to attenuate fibrosis in many tissues including the liver, kidney, pancreas, lung, and heart [35–46]. In ocular tissues, several *in vitro* and *ex vivo* studies have showed the inhibitory effect of Smad7 in fibrosis [36,43,44]. We therefore hypothesized that targeted ectopic overexpression of Smad7 in the cornea may inhibit injury or surgery induced corneal fibrosis. In this study, we demonstrate that loss of Smad7 expression in absence of TGFβ induced a small amount of endogenous α-SMA expression in corneal fibroblasts; however, it was significantly enhanced when the same cells were treated with TGFβ (Fig 1). Gain-of-function of Smad7 by ectopic overexpression led to dramatically reduced TGFβ induced α-SMA expression (Fig 2).

Recombinant AAV vectors have been used successfully for delivering therapeutic genes to provide long-term transgene expression with minimal or no adverse effects [7, 19]. We have previously shown therapeutic efficacy as well as safety of AAV vectors for *in vivo* gene delivery in corneal tissue [6–10,18,19]. Here, we tested the safety and therapeutic efficacy of AAV5-Smad7 gene delivery in an *in vivo* rabbit corneal fibrosis model. Morphological examination through slit-lamp biomicroscopy, stereomicroscopy, and histopathological examination clearly indicate absence of intraocular inflammation, conjunctival hyperemia, ocular discharge, or other ocular pathology in rabbit eyes after AAV5-Smad7 gene delivery alone. Our previous studies compared the transduction efficiency of AAV serotypes for delivering genes in the cornea and found enhanced transgene delivery with AAV5 in the rabbit cornea as compared to other serotypes [6,7,15,47]. Based on our previous findings, we used AAV5 for Smad7 gene delivery. AAV5 vector did not cause any significant apoptosis in the treated corneas as measured by TUNEL assay (Fig 7), nor did it affect the intraocular pressures in the treated eyes (Fig 4). Further CD11b and F4/80 staining indicated the absence of inflammation or infiltration of immune cell in stroma (data not shown). These data indicate that Smad7 gene delivery through AAV5 serotype is safe and has no cytotoxic effect (Figs 3, 4 and 7). This is particularly important since we have observed long-term retention of AAV delivered transgene in the stromal layer of mouse corneas [10].

We observed that the expression of α-SMA and fibronectin were reduced as a result of AAV5-Smad7 gene delivery (Figs 5 and 6) proving its therapeutic potential. However, it should be noted that there are multiple factors that potentially regulate TGFβ signaling cascades relevant to fibrosis. While TGFβ activating mechanisms depend on receptor-activated Smads, SARA, YAP, and others, the association with FKBP12, Smurf1, Ski, etc have regulatory or inhibitory signaling functions [48]. Therefore, in a clinical setting, an approach that incorporates the multiple modalities may have greater therapeutic efficiency. Our previous work has explored alternative targets such as Decorin [8], Id1-4 [49,50] and regulation of epigenetic mechanisms [28], all of which may be contributing factors towards treating corneal haze. Smad7, in particular, has been shown to compete with Smad4 and oligomerize with R-Smads to form heteromeric complex thereby interfering with R-Smad-Smad4 oligomerization [23], thereby disrupting the TGFβ signaling cascade. Earlier studies have demonstrated that epithelial to mesenchymal transition (EMT) plays a critical role in fibrosis [51]. Hence, Smad7 overexpression might lead to repression of EMT leading to downregulation of α-SMA and upregulation of E-cadherin [40]. Therefore, identification of interactions and crosstalk between TGFβ-mediated Smad and non-Smad signaling pathways [48] including proteasomal degradative regulation of Smads or Hippo pathway members may provide novel insights and putative molecular mechanisms underlying corneal fibrosis.

In conclusion, we demonstrate the safety and therapeutic efficacy of targeted AAV5-Smad7 gene therapy to inhibit *in vivo* corneal scarring in this animal model.

## Supporting information

S1 Table Corneal haze on Fantes grading scale. Fantes grading was performed by three independent observers in a masked manner at various times.
(PDF)

S2 Table Intraocular pressure (IOP) in the eyes of naïve, AAV5-naked and AAV5-Smad7 treated animals. IOP were recorded in rabbits using a TONO-PEN at day 0, 7, 14, 21 and 28 days. All IOP measurements were performed between 9-11am to minimize normal diurnal variations in IOP. The data given is an average of three readings.
(PDF)
