# Supplementary material for: Targeted AAV5-Smad7 gene therapy inhibits corneal scarring in vivo
Source: PLoS One. 2017 Mar 24;12(3):e0172928. doi: 10.1371/journal.pone.0172928 (PMC5365107; doi:10.1371/journal.pone.0172928)
Supplement: S1 Table — Fantes grading was performed by three independent observers in a masked manner at various times. (PDF) [file pone.0172928.s001.pdf]

Haze Grading (Fantes Scale)																								
**Observer-1**	**Naïve control**								**PRK + rAAV naked**								**PRK + rAAV-Smad7**							
**Animal**	**1**	**2**	**3**	**4**	**5**	**6**	**Mean**	**SEM**	**1**	**2**	**3**	**4**	**5**	**6**	**Mean**	**SEM**	**1**	**2**	**3**	**4**	**5**	**6**	**Mean**	**SEM**
**Time point**																								
Day 0 (before PRK)	0.0	0.0	0.0	0.0	0.0	0.0	**0.0**	0.00	0.0	0.0	0.0	0.0	0.0	0.0	**0.0**	0.00	0.0	0.0	0.0	0.0	0.0	0.0	**0.0**	0.00
Day 7	0.0	0.0	0.0	0.0	0.0	0.0	**0.0**	0.00	0.8	0.7	0.8	0.8	0.9	0.7	**0.8**	0.03	0.8	0.6	0.6	0.7	0.7	0.6	**0.7**	0.03
Day 14	0.0	0.0	0.0	0.0	0.0	0.0	**0.0**	0.00	1.5	0.9	1.0	1.0	0.9	1.6	**1.2**	0.13	0.9	0.8	0.9	0.9	0.8	0.8	**0.9**	0.02
Day 21	0.0	0.0	0.0	0.0	0.0	0.0	**0.0**	0.00	2.1	2.4	1.8	2.3	3	3	**2.4**	0.20	1.5	1.5	1.6	1.5	1.5	1.5	**1.5**	0.02
Day 28	0.0	0.0	0.0	0.0	0.0	0.0	**0.0**	0.00	2.2	2.4	2.1	2.2	3.5	3.2	**2.6**	0.24	1.2	1.1	1.6	1.2	1.6	1.6	**1.4**	0.10
**Observer-2**	**Negative control 1 (Naïve)**								**PRK + (rAAV naked)**								**PRK + (rAAV-SMad7)**							
	**1**	**2**	**3**	**4**	**5**	**6**	**Mean**	**SEM**	**1**	**2**	**3**	**4**	**5**	**6**	**Mean**	**SEM**	**1**	**2**	**3**	**4**	**5**	**6**	**Mean**	**SEM**
**Time point**																								
Day 0 (before PRK)	0.0	0.0	0.0	0.0	0.0	0.0	**0.0**	0.00	0.0	0.0	0.0	0.0	0.0	0.0	**0.0**	0.00	0.0	0.0	0.0	0.0	0.0	0.0	**0.0**	0.00
Day 7	0.0	0.0	0.0	0.0	0.0	0.0	**0.0**	0.00	0.8	0.7	0.8	0.8	0.9	0.7	**0.8**	0.03	0.8	0.6	0.6	0.7	0.7	0.6	**0.7**	0.03
Day 14	0.0	0.0	0.0	0.0	0.0	0.0	**0.0**	0.00	1.3	0.9	1.0	1.0	1.6	0.8	**1.1**	0.12	0.9	0.8	0.9	0.9	0.8	0.8	**0.9**	0.02
Day 21	0.0	0.0	0.0	0.0	0.0	0.0	**0.0**	0.00	1.5	2.0	2.1	2.3	2.3	2.9	**2.2**	0.19	1.3	1.4	1.6	1.5	1.6	1.5	**1.5**	0.05
Day 28	0.0	0.0	0.0	0.0	0.0	0.0	**0.0**	0.00	1.9	2.0	3.5	2	3.1	2.5	**2.5**	0.27	1.1	1.5	1.2	1.6	1.6	1.2	**1.4**	0.09
**Observer-3**	**Negative control 1 (Naïve)**								**PRK + (rAAV naked)**								**PRK + (rAAV-SMad7)**							
	**1**	**2**	**3**	**4**	**5**	**6**	**Mean**	**SEM**	**1**	**2**	**3**	**4**	**5**	**6**	**Mean**	**SEM**	**1**	**2**	**3**	**4**	**5**	**6**	**Mean**	**SEM**
**Time point**																								
Day 0 (before PRK)	0.0	0.0	0.0	0.0	0.0	0.0	**0.0**	0.00	0.0	0.0	0.0	0.0	0.0	0.0	**0.0**	0.00	0.0	0.0	0.0	0.0	0.0	0.0	**0.0**	0.00
Day 7	0.0	0.0	0.0	0.0	0.0	0.0	**0.0**	0.00	0.8	0.7	0.8	0.8	0.9	0.7	**0.8**	0.03	0.8	0.6	0.6	0.7	0.7	0.6	**0.7**	0.03
Day 14	0.0	0.0	0.0	0.0	0.0	0.0	**0.0**	0.00	1.1	1.5	1.7	1.1	2.2	2.0	**1.6**	0.19	0.9	0.8	0.9	0.9	0.8	0.8	**0.9**	0.02
Day 21	0.0	0.0	0.0	0.0	0.0	0.0	**0.0**	0.00	1.5	1.6	1.8	2.2	2.8	3.1	**2.2**	0.27	1.2	1.4	1.6	1.5	1.5	1.2	**1.4**	0.07
Day 28	0.0	0.0	0.0	0.0	0.0	0.0	**0.0**	0.00	2	1.8	1.9	3.5	2.9	3.1	**2.5**	0.30	1	1.5	1.8	1.2	1.5	1.6	**1.4**	0.12
