# Supplementary material for: Targeted AAV5-Smad7 gene therapy inhibits corneal scarring in vivo
Source: PLoS One. 2017 Mar 24;12(3):e0172928. doi: 10.1371/journal.pone.0172928 (PMC5365107; doi:10.1371/journal.pone.0172928)
Supplement: S2 Table — IOP were recorded in rabbits using a TONO-PEN at day 0, 7, 14, 21 and 28 days. All IOP measurements were performed between 9-11am to minimize normal diurnal variations in IOP. The data given is an average of three readings. (PDF) [file pone.0172928.s002.pdf]

IOP Measurement																										
	Naïve control									PRK + rAAV naked									PRK + rAAV-Smad7							
Animal	1	2	3	4	5	6	Mean	SEM		1	2	3	4	5	6	Mean	SEM		1	2	3	4	5	6	Mean	SEM
Time point																										
Day 0 (before PRK)	9.0	10.0	11.0	9.0	10.0	10.0	**9.8**	0.31		14.0	8.0	9.0	9.0	9.0	11.0	**10.0**	0.89		14.0	10.0	8.0	10.0	11.0	13.0	**11.0**	0.89
Day 7	11.0	12.0	13.0	9.0	9.0	11.0	**10.8**	0.65		14.0	10.0	8.0	12.0	11.0	12.0	**11.2**	0.83		8.0	8.0	14.0	8.0	10.0	11.0	**9.8**	0.98
Day 14	9.0	11.0	10.0	12.0	13.0	10.0	**10.8**	0.60		13.0	9.0	9.0	9.0	10.0	9.0	**9.8**	0.65		9.0	12.0	12.0	11.0	10.0	12.0	**11.0**	0.52
Day 21	10.0	11.0	12.0	9.0	8.0	10.0	**0.0**	0.58		9.0	8.0	10.0	12.0	12.0	10.0	**10.2**	0.65		13.0	12.0	10.0	11.0	9.0	11.0	**11.0**	0.58
Day 28	11.0	10.0	8.0	9.0	10.0	11.0	**0.0**	0.48		15.0	15.0	8.0	8.0	8.0	11.0	**10.8**	1.40		15.0	8.0	15.0	8.0	8.0	12.0	**11.0**	1.41
